# Coarse‐graining bacteria colonies for modelling critical solute distributions in picolitre bioreactors for bacterial studies on single‐cell level

**DOI:** 10.1111/1751-7915.12708

**Published:** 2017-04-03

**Authors:** Christoph Westerwalbesloh, Alexander Grünberger, Wolfgang Wiechert, Dietrich Kohlheyer, Eric von Lieres

**Affiliations:** ^1^ Institute of Bio‐ and Geosciences, IBG‐1: BiotechnologyForschungszentrum JülichJülich52425Germany

## Abstract

Microfluidic single‐cell bioreactors have found widespread application to investigate growth and gene expression of microbial model organisms, but yet there are few attempts to systematically characterize different design and cultivation concepts. Quantitative measurements of critical solute concentrations, e.g. limiting nutrients, are not yet feasible within the typical volumes in the range of picolitres. A way to gain new insights about the mass transport within those volumes is by simulation, but the complex geometry resulting from the multitude of cells within a colony leads to time and resource consuming computational challenges. In this work, six different concepts for the model representation of cellular microcolonies within microfluidic monolayer growth chamber devices are compared. The Gini coefficient is proposed as new measure for inhomogeneity within cellular colonies. An example cell colony is represented by a single point source, a cylindrical volume with homogeneous reaction rates with and without adjusted diffusion coefficient, as point sources for each single cell and as rod‐shaped, diffusion blocking, three‐dimensional cells with varying shapes. Simulated concentration profiles across the chambers depended strongly on the chosen cell representation. The representation with the lowest degree of abstraction, three‐dimensional cells, leads to complex geometries and high computational effort, but also gives a conservative and therefore preferable estimate for the cultivation conditions within a given cultivation chamber geometry. Interestingly, the cylindrical volume with adjusted diffusion coefficient gives similar results but requires far less computational effort. Therefore, it is proposed to use the three‐dimensional cells for detailed studies and to determine parameters for the cylindrical volume with adjusted diffusion coefficient, which can then be used for experimental design, screening of parameter spaces, and similar applications.

## Introduction

**Figure 1:**
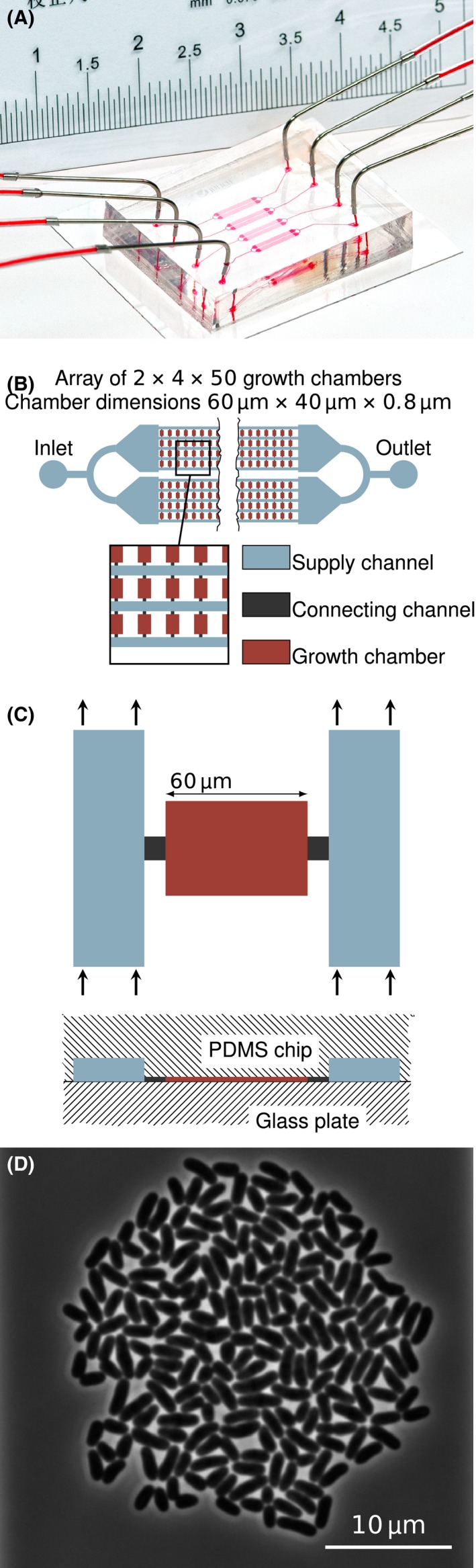
Microfluidic single‐cell cultivation platform used as example for this study: (A) Picture of the microfluidic chip, (B) Structure of one chamber array, (C) Single cultivation chamber, (D) Image of a *Corynebacterium glutamicum* colony after 23.3 h of cultivation comprising approx. 200 cells.

Recent findings suggest a significant influence of cell‐to‐cell heterogeneity on a wide variety of important biological phenomena, including biofilm formation, antibiotic resistance, microbiome stability, and product yields and robustness of industrial bioprocesses (Lara *et al*., 2006; Haselgrübler *et al*., 2014; Rusconi *et al*., 2014). Therefore, novel cultivation techniques are required to investigate cellular heterogeneity while distinguishing extrinsic heterogeneity, e.g. by nutrient gradients, from intrinsic heterogeneity, e.g. by stochasticity of biochemical reactions. Here, microfluidic single‐cell cultivation offers various new opportunities for research of microorganisms on single‐cell level (Liu *et al*., 2012; Grünberger *et al*., 2014; Mehling and Tay, 2014). A major advantage is the micrometre length scale of the devices, enabling the cultivation of observed microorganisms at defined environments and with good spatiotemporal resolution (Fig. 1).

Especially two‐dimensional cultivation chamber systems, which prohibit cell colony growth in the third dimension, offer a good compromise between environmental control with regard to the cultivation medium, i.e. constant nutrient and low product and by‐product concentration, observable colony size and the possibility to automatically analyse image data and evaluate results (Dusny *et al*., 2015; Grünberger *et al*., 2015). They are typically characterized by long and deep supply channels with a constant stream of fresh cultivation medium (Fig. 1B) and interjacent flat cultivation chambers (Fig. 1C), which allow cell colony growth only in monolayers (Fig. 1D). These monolayer growth chambers have already found application in the investigation of streaming patterns caused by cell growth (Mather *et al*., 2010), in‐depth analysis of cell growth, size and physiology (Taheri‐Araghi *et al*., 2015; Hashimoto *et al*., 2016; Wallden *et al*., 2016), heterogeneity studies of growth, division and spontaneous stress response (Grünberger *et al*., 2013, 2015), dynamics and distribution of spontaneous prophage induction (Helfrich *et al*., 2015b), the history dependence of stress reactions (Mathis and Ackermann, 2016), and metabolite production (Mustafi *et al*., 2012, 2014).

In comparison to laboratory‐ and large‐scale bioreactors, novel single‐cell systems are often not fully characterized. While such devices are typically well characterized regarding fluid flow, the availability of nutrients is not regularly considered in detail. However, local concentrations of solutes are important as they determine the environmental conditions of the microorganisms at their locations. Many microfluidic studies are performed with media containing a high surplus of all necessary nutrient compounds, so any possible gradients can be neglected. This is independent of the media type, i.e. complex or defined. Nonetheless, experiments under limiting environmental conditions can be important for studying fundamental microbiological phenomena such as growth, evolution or product formation. Due to the length scale in the micrometre range and corresponding small volumes of several picolitres per cultivation chamber, it is currently very challenging to directly measure concentration changes of nutrients or oxygen availability, which are in turn usually neglected, within the devices. Computational simulation offers a way to investigate the fluid flow and mass transport within microfluidic devices without direct measurements of flow velocities or local concentrations. In a previous study, simulations were performed to investigate concentration gradients within bacterial microcolonies (Westerwalbesloh *et al*., 2015). It was found that, depending on the nutrient concentration and colony size, the microorganisms have a strong influence on local conditions and can create significant gradients, i.e. different nutrient availability across the bacterial colonies, where cells close to the centre showed significantly slower growth than cells at the outer boundary of the colony. These results emphasize the need for quantification of mass transport phenomena to help design and operate microfluidic devices in a well‐defined and reproducible manner.

**Figure 2:**
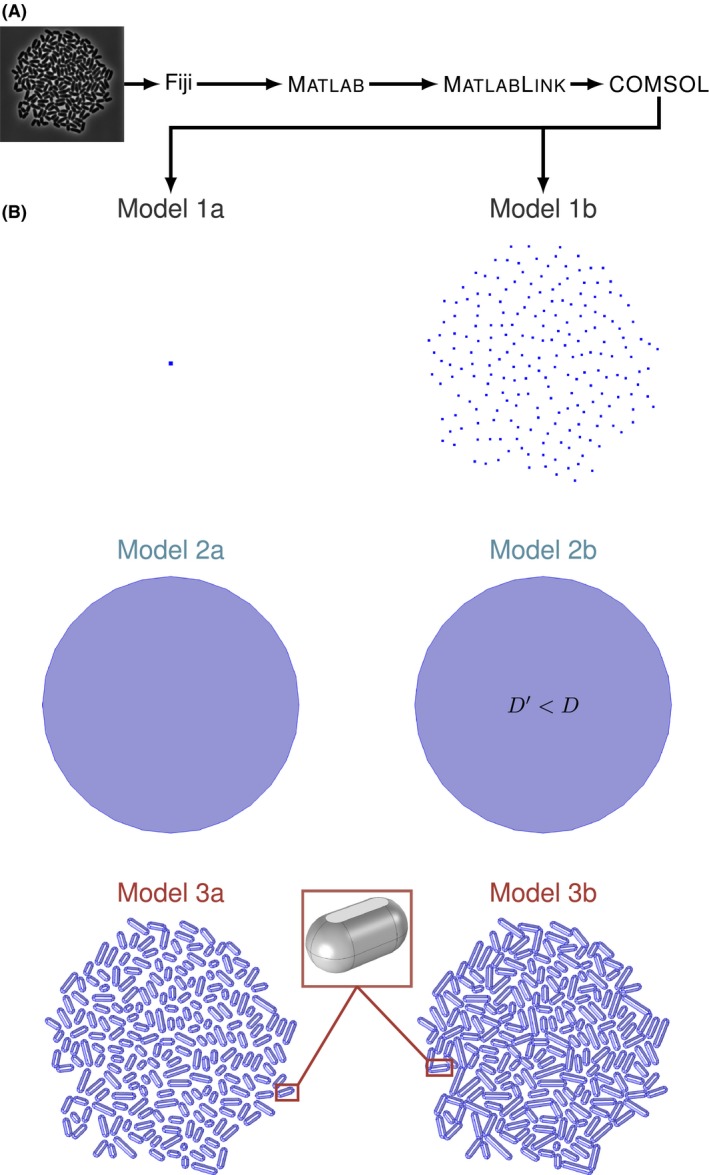
(A) Workflow for geometry creation from experimental data. (B) Cell colony representations: colony point source model (Model 1a), colony with cells as point sources (Model 1b), colony as homogeneous volume (Model 2a), colony as homogeneous volume with adjusted diffusion coefficient (Model 2b), cells as 3D bodies (Model 3a) and cells with elongated 3D bodies (Model 3b). Magnification (red rectangle) shows the three‐dimensional representation of a single cell.

For a simulation, which does not neglect the influence of the microorganisms on their environment, the cell colonies or single cells have to be represented adequately. Apart from the metabolic model, which directly contributes to the equations describing mass transport, for models with spatial resolution the cells and colonies also have to be represented in a geometrical manner. In this study, different approaches for geometric representations of cellular colonies are investigated for their use in simulation of single‐cell level cultivation devices (Fig. 2B). Simple approaches are to introduce zero‐dimensional point sources at discrete locations, either a cumulative source for a whole colony or one for each cell (Model 1a and Model 1b). An alternative is to calculate an average reaction rate per cultivation volume and add a homogeneous term to the respective equations describing the advection and diffusion of solutes (Model 2a). An adjusted effective diffusion coefficient is also introduced within the area of the colony to reflect hindered mass transfer (Model 2b). Furthermore, cells are modelled explicitly as three‐dimensional geometric objects, based on the assumption that microorganisms impair mass transfer of nutrients like glucose via reduced diffusion of molecules through their cell membranes (Model 3a and Model 3b), as it has been reported for several macroscopic experiments (Wood and Whitaker, 2000). This is a computationally expensive method, as it leads to complex geometries requiring more mesh elements for the computational fluid dynamics (CFD) simulation. As the computational difficulty (Table S1), time consumption, and results of the calculations vary to a high degree with the chosen geometric cell representation, the different approaches were evaluated systematically for microfluidic monolayer growth chamber systems (Fig. 1). Within this work, a colony of *Corynebacterium glutamicum* at five different time points and thus number of cells was used as a case study (Grünberger *et al*., 2015).

## Results and discussion

The six different models were used to estimate concentration profiles across five example colonies of *C. glutamicum* (Fig. 2B). The results are compared in terms of the calculated concentration profiles, the average relative uptake rates as a measure for differences between ideal and actual conditions, and the Gini coefficient as a measure for differences between single cells (Section Evaluation). Both, relative uptake rate and Gini coefficient, have to be minimized below acceptable values to exclude effects based on differences in nutrient supply from experimental results. This comparison will help choosing the right geometric representation for future modelling applications. Here, we focus on two medium nutrient concentration levels, the lowest and the highest simulated values (0.05 and 50 mmol l^−1^). Results for other simulated medium nutrient concentrations (0.5 and 5 mmol l^−1^) are shown in the supplementary information (Figs S3–S7).

### Relative uptake rate

**Figure 3:**
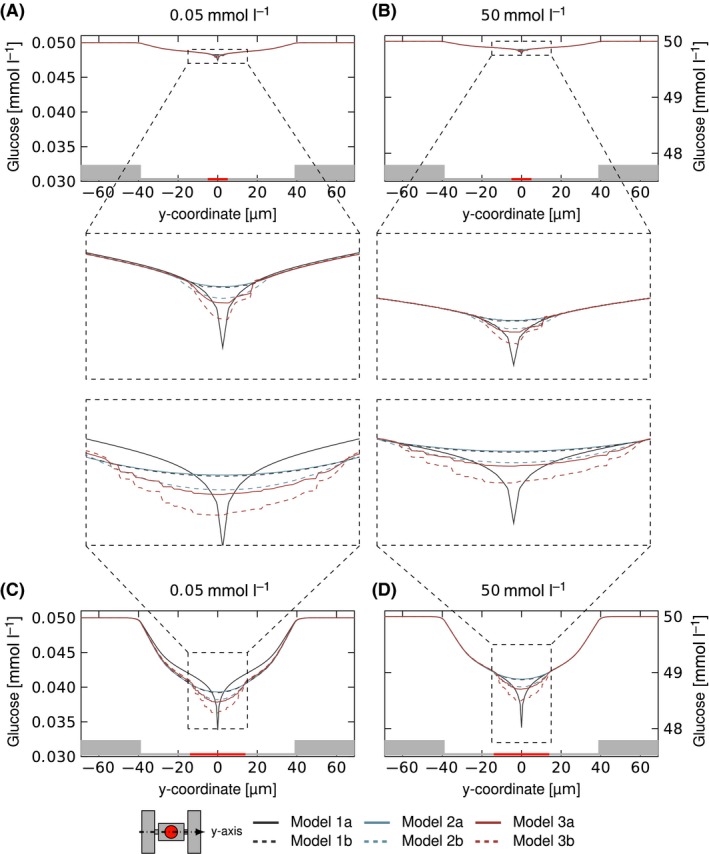
Nutrient distribution within microcolonies based on simulations with different coarse‐graining concepts. The graphs and respective zoom‐outs show the glucose concentration along the *y*‐axis through the centre of the chamber/colony in mmol l^−1^ for the colony with 20 cells (A, B) and 200 cells (C, D) and two medium glucose concentrations of 0.05 mmol l^−1^ (A, C) and 50 mmol l^−1^ (B, D).

The relative uptake rate is a measure to compare conditions with ideal nutrient supply and the actual nutrient availability. Therefore, it is directly influenced by the concentration difference between the growth chamber and the supply channels. In Fig. 3, the concentration profiles along the *y*‐axis are shown. In the supply channels, nutrient concentrations are equal to pure growth medium, as can be seen for the *y*‐coordinates below −39 μm and above 39 μm. It is assumed the flow rates are high enough to neglect the influence of the cellular uptake on the concentration in the supply channels.

**Figure 4:**
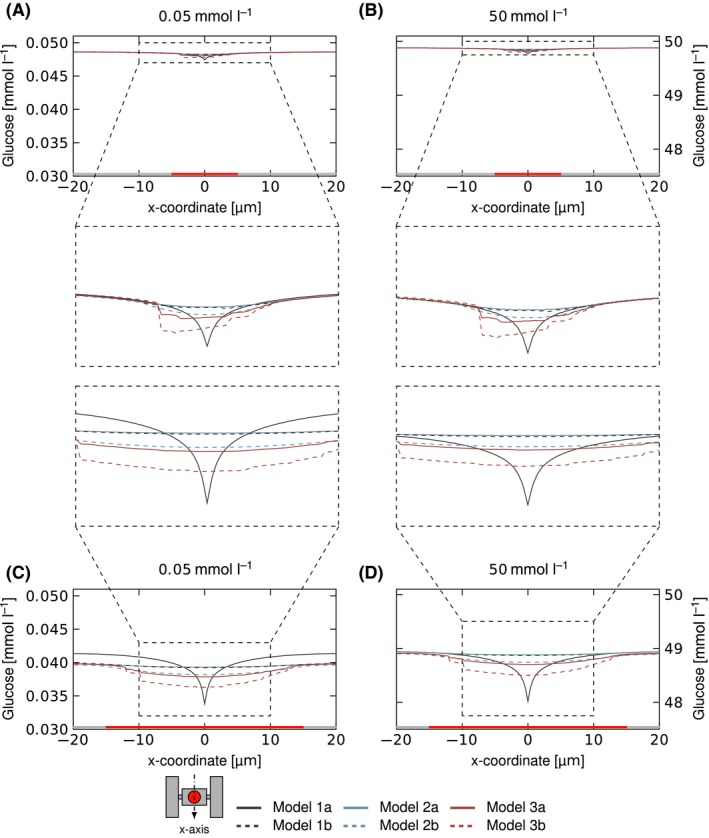
Nutrient distribution within microcolonies based on simulations with different coarse‐graining concepts. The graphs and respective zoom‐outs show the glucose concentration along the *x*‐axis through the centre of the chamber/colony in mmol l^−1^ for the colony with 20 cells (A, B) and 200 cells (C, D) and two medium glucose concentrations of 0.05 mmol l^−1^ (A, C) and 50 mmol l^−1^ (B, D).

Within the connecting channels and inside the area of the chamber not occupied by the colony, a gradient with a decreasing concentration towards the chamber centre is visible. In this area, there are no significant differences between the models with the exception of Model 1a, where the gradient is less steep and the concentrations are higher, especially for the low medium nutrient concentration (0.05 mmol l^−1^, grey line in Figs 3C and 4C). This is a result of the accumulation of the total colony uptake within a single point at the centre of the colony. The steeper gradient close to the centre results in a low concentration at this point, which in turn lowers the uptake for nutrient concentrations close to the half velocity constant of the Monod kinetic. This lower overall colony uptake for Model 1a causes the higher concentration values and less steep gradients outside of the colony area and explains why they occur especially for the combination of lowest medium nutrient concentration and biggest colony.

**Figure 5:**
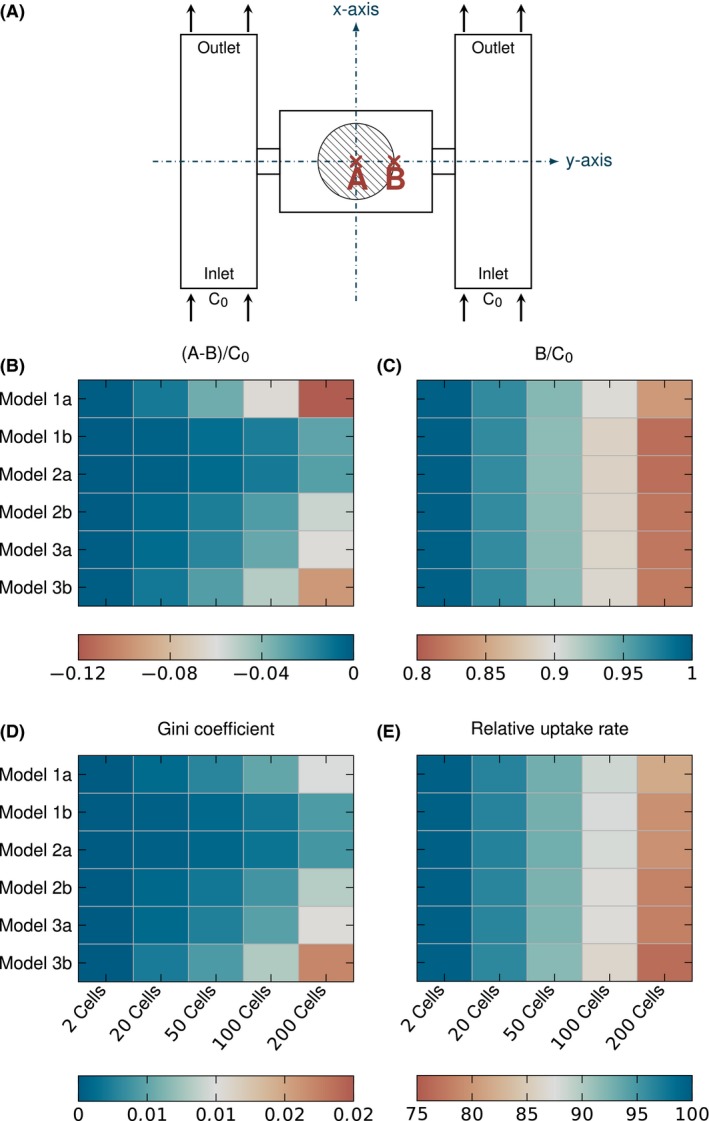
Schematic drawing of the axis and the positions *A* and *B* (A), the concentration difference across the colony *A *− *B* normalized to inlet concentration *C*_0_ (B), the concentration at the colony boundary *B* normalized to *C*_0_ (C), the Gini coefficient (D) and the average relative uptake rate (E) for each model and colony size and the nutrient concentration *C*_0_ of 0.05 mmol l^−1^.

**Figure 6:**
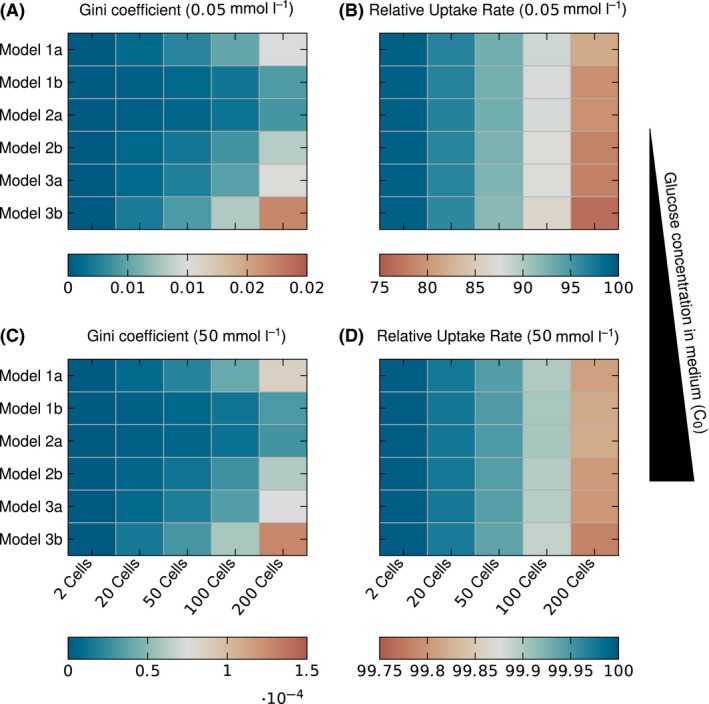
The Gini coefficient for inlet concentrations of *C*_0_ = 0.05 mmol l^−1^ (A) and *C*_0_ = 50 mmol l^−1^ (C) and the average relative uptake rate for *C*_0_ = 0.05 mmol l^−1^ (B) and *C*_0_ = 50 mmol l^−1^ (D).

To quantify the gradients directly, single points *A* and *B* are introduced as representative of the concentrations at the centre and boundary of the colony (Fig. 5A). *B* has been normalized with respect to nutrient concentration of pure medium (*C*_0_) (Fig. 5C). While this is already a simple way to compare expected and actual conditions, it is still not easy to estimate the effects of the gradient on measured growth rates or compare different medium nutrient concentration levels with each other. The proposed alternative, the relative uptake rate, shows a pattern very similar to the normalized concentrations at the boundary of the colony (*B*/*C*_0_) (Fig. 5E). However, there are small differences especially visible for 200 cells. Growing colonies have an increasing amount of cells and biomass, resulting in a bigger overall uptake rate, which in turn leads to stronger gradients and lower concentrations even at the outer boundary of the colony. The differences can be explained by the influence of gradients within the colony on the average colony uptake, because cells close to the centre will have a lower relative uptake rate for lower nutrient concentrations. Gradients within the colony influence the concentration at the boundary of the colony, *B*, less, which therefore takes similar values for most models but Model 1a, where the concentration gradient generally is different. The relative uptake rate is not very sensitive towards model choice, but shows a strong correlation with medium nutrient concentration (Fig. 6). Figure 6D shows qualitatively similar results for a high nutrient concentration but on a very different scale. A small change in concentration at limiting conditions, for example, will have a stronger effect on nutrient uptake and growth rate, than the same change at nutrient surplus. The relative uptake rate shows good agreement with the simulated concentration profiles, but it is easy to apply and interpret for a wide range of concentrations and metabolic models. Furthermore, it includes basic information about the metabolism's dependence on concentration levels and its sensitivity towards absolute concentration changes. It is also independent of the chosen geometry and design principles for the investigated microfluidic device and thus simplifies comparisons.

### Gini coefficient

The Gini coefficient (Definition see section Evaluation) describes cell‐to‐cell heterogeneity and therefore depends on the concentration gradients within the area of the colony (Figs 3 and 4, colony area highlighted in red and zoom‐out plot). The concentration profiles within the area of the colony show strong variation depending on the model choice; especially, Model 1a (grey line) shows a very steep gradient and the lowest extremum of nutrient concentration, as explained earlier. Model 1b (dashed grey line) and Model 2a (blue line) both distribute the uptake over the colony area and show nearly identical concentration profiles. Model 2b (dashed blue line), Model 3a (red line) and Model 3b (dashed red line) all hinder diffusion, different from the other models. Therefore, the concentration profiles within the colony show stronger gradients, indicating a bigger difference between the cells at the boundary and within the centre of the colony than for the models allowing unhindered diffusion. The concentration profile for Model 2b and Model 3a show similar gradients. Both Model 3a and Model 3b show irregular profiles due to the inhomogeneity introduced by the placement of individual cells.

The normalized concentration difference between colony boundary and centre, (*A* − *B*)/*C*_0_, quantifies the gradient across the colony (Fig. 5B). The proposed alternative, the Gini coefficient, correlates well with those numbers (Fig. 5D). The biggest difference is that, while the concentration difference shows worst values for Model 1a, the Gini coefficient is worst for Model 3b. Here, the reason is that the Gini coefficient and the relative uptake rate are calculated with concentrations at the positions of the cells, no matter which model is used. Otherwise a heterogeneity measure cannot be calculated for colony‐level models like Model 1a or Model 2a. Many of the cell positions are in areas with favourable conditions in Model 1a, so the Gini coefficient for this model has better values despite a strongest overall gradient.

The Gini coefficient grows with the colony size, as is expected as the cells in the centre will always experience lower concentrations than at the boundary for diffusive mass transport within the colony. It also corresponds well to the concentration profiles seen in Figs 3 and 4, as Model 1b and Model 2a show the lowest heterogeneity and gradients, followed by Model 2b, Model 3a and Model 1a. Model 3b describes strongly hindered diffusion, as the long cells restrict diffusion paths, and leads to the high inhomogeneity. Figure 6 shows the Gini coefficients for the lowest (0.05 mmol l^−1^, Fig. 6A) and highest (50 mmol l^−1^, Fig. 6C) medium nutrient concentration. Both show the same pattern, but the coefficients of 50 mmol l^−1^ are on a different scale two orders of magnitude lower than for 0.05 mmol l^−1^, in good agreement with the Monod kinetics.

A major advantage of the Gini coefficient is that there is no need to determine the best points of measurement for the gradient, which is difficult for complex colony geometries that differ from the simple circular shape used in this study. Similar to the relative uptake rate, the Gini coefficient is independent from specific design choices and can be applied on a broad range.

### Cell model comparison

The Gini coefficient and the relative uptake rate are useful measures to quantify the inhomogeneity and general conditions in observed cell colonies, as they correlate well with the simulated concentration profiles (Figs 3, 4, 5). Therefore, they can be used to compare the different cell geometry representations with each other.

The chosen model has a strong influence on the results, with three‐dimensional cells representing the model with the highest level of detail and a conservative estimate, as this leads to less favourable conditions in the simulation. The importance of the cell shapes and colony density can be seen in the differences between Model 3a and Model 3b, where the uptake rate per cell is identical, but Model 3b has longer cell bodies. Both models lead to complex geometries, which cause in turn high computational effort for the solution (Table S1). The geometry creation also requires positions, angles and sizes of all cells, information which might be difficult to obtain in early design stages or without good image recognition software.

Model 2b is a good method to simplify the difficult geometry creation and resource‐intensive calculation caused by detailed geometries and can cover many different cases by adjustment of the diffusive hindrance using *ε*_*σ*_. The theory behind the adjusted diffusion coefficients is based on regularly spaced cylinders, which are significantly smaller than the area over which the hindered diffusion is observed. This is usually not fulfilled for the investigated microfluidic devices and colonies. Therefore, it is recommended to take into account the actual cellular geometry. Nonetheless, the results of Model 2b are very comparable to Model 3a, so that it is an effective tool to limit required computational resources, e.g. for parameter studies, if *ε*_*σ*_ is chosen carefully or based on more thorough simulations, e.g. Model 3a.

The calculated results show that concentration gradients within the colony can be expected to be small enough for most experiments to assume homogeneous conditions independent of the chosen cell model, but depending on the experimental set‐up it is advisable to verify this assumption. It is important to keep in mind the Monod half velocity constant of 4.5 mmol l^−1^ chosen for the calculations is of the same order of magnitude or bigger than the low glucose concentrations in this study. A smaller half velocity constant results in decreased influence of the concentration gradient on the cellular uptake.

### Conclusions

In this study, we have introduced for the first time a cellular Gini coefficient for the quantification of inhomogeneity within a cellular colony for combinations of microfluidic design and operating parameters. The Gini coefficient has been shown to be a useful measure, as it condenses the concentration gradient results to one easily interpretable number. The relative uptake rate provides additional information about the comparability of the conditions within the chambers and growth medium composition. In the case investigated in this study, a Gini coefficient below 0.7% and a relative uptake rate above 96% were sufficient to ensure a maximum difference in uptake rate between two cells of 5% and a maximum difference to the theoretical optimum uptake rate of 5% for all models except Model 1a, which has not been taken into account as it is regarded as too coarse‐grained.

Two representations, a model where cells are represented by a homogeneous volume with adjusted diffusion coefficient (Model 2b), and a model where cells are represented by 3‐dimensional objects (Model 3a), stand out for future modelling studies. Model 3a promises to be a good approximation, as it models the observed geometry closely and many macroscopic experiments have reported hindered diffusion by cells. It also provides a conservative estimate until future experiments determine the exact influence of cells on diffusion in microfluidic devices, as it consistently leads to high estimates for the inhomogeneity and gradients across the cellular colonies. As the complex geometry requires many mesh elements and significantly higher computational effort to solve than the other, simpler geometries, Model 2b is an attractive alternative to estimate conditions within colonies. It can be used for screening and preliminary design studies due to its reduced computational requirements.

The methods described in this study lay a foundation to characterize microfluidic experiments as well as define minimal requirements to guarantee homogeneous and well‐defined conditions across observed microcolonies, e.g. for cell‐to‐cell heterogeneity characterization. First, experiments have already shown gradients across cell cultures for cultivation with low concentrations of protocatechuic acid as sole carbon source (Westerwalbesloh *et al*., 2015). A better specification of model parameters gained from future experiments, e.g. metabolic kinetics, will certainly help to improve the predictive quality of the model.

Similar devices have been employed for investigating other organisms, e.g. *Escherichia coli* or yeast, so that the results of this study are expected to help designing and understanding a wide variety of microfluidic devices. In this study, glucose was used as example solute, but the presented methods can be applied to other nutrients or products as well. The Gini coefficient and the relative uptake rate are potentially also useful for other extrinsic influences, e.g. dissolved oxygen concentration, temperatures and signalling molecules.

## Experimental procedures

### Computational model

The general model structure and assumptions are identical to the ones previously used (Westerwalbesloh *et al*., 2015). After the flow profile within one chamber and adjacent supply channels has been calculated, the diffusion equation is solved in combination with a model for the cell metabolism to yield concentration profiles for the solute nutrients. The chamber geometry with the supply channels is shown in Fig. 1C. However, the geometric representation of the microorganisms is varied, resulting in different model geometries which are all meshed and solved independently from each other (see Fig. 2). A basic modelling assumption of Westerwalbesloh *et al*. (2015) has been that the conditions in each growth chamber are independent of the chamber's position on the chip. Therefore, it is sufficient to model one chamber with the adjacent channels in detail (Fig. S1). The mass transfer of the example nutrient glucose takes place in the fluid within the chambers and in the channels (Fig. 1B). The highest studied concentration for glucose is 50 mmol l^−1^, and therefore, the solute can be neglected in the calculation of the liquid velocity field. Consequently, the mass transfer equations for the solutes are solved separately after solving the Stokes equations for the velocity field. The model is solved using comsol multiphysics® 5.2 (COMSOL AB, 2016). The mesh is created using the ‘Physics‐controlled mesh’ option using the ‘Extra fine’ setting. Here, boundary layers are added along no‐slip boundaries and a free tetrahedral mesh fills the space between the objects. A finer mesh did not show significant differences for the solution so that the element size is assumed to be sufficiently small (Table S2). Linear functions are used to calculate the concentration, velocity and the pressure profiles.

#### Cell metabolism

*Corynebacterium glutamicum* serves as model organism for this work, but it is reasonable to expect that the methods can be transferred to similarly shaped organisms like *E. coli* or other round/nearly spherical cells of comparable dimensions. The biovolume of the cells is estimated assuming a cell can be seen as cylinder with circular domes at each end. The radius of the cells is approximated as constant (*R*_cell_ = 0.42 μm). Then, the determined area *A*_cell_ (in m^2^) from experimental time‐lapse images can be used to calculate the overall length *L*_cell_ (in m) and the cellular biovolume *V*_cell_ (in m^3^) for each individual cell:

(1) $$L_{\mathrm{cell}}=2R_{\mathrm{cell}}+\frac{A_{\mathrm{cell}}-\pi R_{\mathrm{cell}}^{2}}{2R_{\mathrm{cell}}}$$

(2) $$V_{\mathrm{cell}}=\frac{4}{3}\pi R_{\mathrm{cell}}^{3}+\left(L_{\mathrm{cell}}-2R_{\mathrm{cell}}\right)\pi R_{\mathrm{cell}}^{2}.$$

The cell metabolism is represented using the Monod model. In the case of glucose, it can be assumed that no substrate inhibition takes place for concentrations up to 50 mol m^−3^ (Khan *et al*., 2005). The Monod equation is similar to the Michaelis–Menten enzyme kinetic and has been used as an empirical model for the behaviour of cell populations. It is expressed by the following equation (Monod, 1949):

(3) $$\text{Upt}={\text{Upt}}^{\max}\cdot \frac{c}{K+c}.$$

Here Upt (in mmol/(Ls)) is the uptake of nutrients, depending on the concentration *c* (in mmol/L), the maximum uptake rate Upt^max^ and the Monod constant *K*. The value of *K* is given for glucose with 4.5 mmol/L (Wendisch *et al*., 2000). The maximum uptake rate Upt^max^ is calculated for each cell individually depending on the biovolume *V*_cell_ (Eq. (2)). For an uptake rate of 2.08 μmol_GLC_/(g_CDW_ s) glucose per gram cell dry weight (Lee *et al*., 1998) and 8 × 10^−7^ $m_{\mathrm{Cellvolume}}^{3}/g_{\mathrm{CDW}}$ as biovolume per cell dry weight (Rönsch *et al*., 2003), an uptake rate of 2.6 mmol_GLC_/(*L*_Cellvolume_ s) can be estimated.

#### Computational fluid dynamics

The velocity field is calculated in steady state because the microfluidic devices are typically operated under constant conditions for several hours, which is far longer than the time required for a flow profile to develop at low Reynolds numbers. Equations (4) and (5) are the Stokes equations used for the model. The assumption of Stokes flow is valid as the highest Reynolds number within the simulated region occurs in the supply channels with roughly *Re* = 0.02 and is therefore sufficiently smaller than one.

(4) $$\;0=-\nabla p+\mu \nabla^{2}u$$

(5) ∇·u=0.

Here, **u** (in m s^−1^) denotes the velocity vector, ∇ is the gradient operator, *ρ* the density, *p* the pressure (in Pa) and *μ* the viscosity (Deen, 1998). The modelled device is operated close to atmospheric pressure and at 303.15 K, as for the cultivation of *C. glutamicum*. The density of water *ρ* is 995.6 kg m^−3^, and the viscosity *μ* is 7.97 × 10^−4^ Pa s (Comesaña *et al*., 2003). Due to the small height differences within the geometry, the influence of gravitational force is neglected. The fluid within the channels and chambers has mostly properties close to water, especially when it is a growth medium like CGXII, which consists of water with up to 4% glucose and other nutrients (Unthan *et al*., 2014). As the other nutrients are supplied in smaller amounts, the liquid is modelled isothermal, incompressible and Newtonian with the density and viscosity of water.

The flow into the channel from upstream is given approximately by the average velocity in channel direction. The comsol ^®^ laminar inflow feature is used to simulate a fully developed laminar flow profile at the inlet. The average inlet velocity is calculated from the flow rate divided by the channel cross section. This leads to an average inlet velocity of 1.11 × 10^−3^ m s^−1^ for the flow rate of 200 nl min^−1^ at the chip inlet.

Pressure is used as outlet boundary condition and is set to 0 Pa, meaning all calculated pressures are in relation to the unknown pressure at that point. The operating pressure is not significantly different from atmospheric pressure, and therefore, no additional information about the system could be gained from the absolute value. The polydimethylsiloxane (PDMS) and glass walls are assumed to be impermeable to the liquid and the no‐slip condition is used (Bocquet and Barrat, 1994).

#### Mass transfer

Fick's law of diffusion and binary aqueous diffusion coefficients are used for the mass transfer calculations. With a maximum concentration of 50 mmol l^−1^ of the example solute glucose, it is still reasonable to assume that each solute molecule mainly interacts with water. Equation (6) is the resulting stationary conservation equation (Deen, 1998).

(6) $$u\cdot \nabla c=D\nabla^{2}c.$$

Here, *c* denotes the concentration of a critical solute, e.g. glucose, and *D* the binary diffusion coefficient in water. **u** is the local velocity vector which is calculated using Eqs. (4) and (5).

The concentrations of solutes at the inlets of the supply channels are set to the one of pure growth medium in accordance with the assumption that the changes of concentration over the length of the supply channels can be neglected. The nutrient concentration in the supplied medium is varied to cover a wide range of nutrient to cellular uptake ratios: 0.05, 0.5, 5 and 50 mmol l^−1^.

The relation between the advective mass transfer and the diffusive mass transfer can be expressed by the Péclet number Pe. The Pe value for the supply channels is above 10, so that at the outlet the diffusive mass transfer can be neglected in comparison with the advective mass transfer (Deen, 1998). It is assumed that glass and PDMS are impermeable for the investigated solutes and absorption as well as adsorption can be neglected. The diffusion coefficient in water at 30°C for the investigated nutrient glucose has been found to be 5.4 × 10^−10^ m^2^ s^−1^ (Gladden and Dole, 1953).

#### Evaluation

Two additional measures are used apart from the calculated concentration profiles to help characterizing the combination of cellular metabolism and microfluidic device. The Gini coefficient is a measure for inhomogeneity, originally developed in economics to describe income or wealth inequality of populations. It has been proposed for several alternative applications, e.g. to rate the selectivity of inhibitors (Graczyk, 2007), resource inequality between neighbourhoods (Druckman and Jackson, 2008), recruitment inequality in clinical trials (Haidich and Ioannidis, 2004) or seasonal variation in environmental radon gas (Groves‐Kirkby *et al*., 2009). A new application of the Gini coefficient is the description of the inhomogeneity of nutrient uptake rates across a cellular colony. Here, the nutrient uptake is interpreted as an equivalent of the income for each individual cell. Equation (7) describes how the Gini coefficient is calculated for an unordered set of data (Druckman and Jackson, 2008). In this case, *n* is the number of cells, *η* the average of cellular nutrient uptake and *y*_*i*_ and *y*_*j*_ the nutrient uptake of the *i*th and *j*th cell. The nutrient uptake has been normalized with the maximum uptake rate for each cell, where *K* is the Monod half velocity constant and *c*_*i*_ the nutrient concentration at the position of the cell.

(7) $$\text{Gini}=\frac{1}{2n^{2}\eta }\sum_{i=1}^{n}\sum_{j=1}^{n}\left|y_{i}-y_{j}\right|$$

(8) $$y_{i}=\frac{c_{i}}{c_{i}+K}.$$

The Gini coefficient has a maximum of (*n* − 1)/*n* for one cell taking up all the nutrient and the others none, a level of inequality that is unlikely to be reached within a single microcolony, and a minimum of zero for total equality.

Another measure is the relative uptake rate (Westerwalbesloh *et al*., 2015). Generally, the good environmental control within microfluidic devices is used to cultivate cells close to pure growth medium conditions. Therefore, the relative uptake rate, which is calculated by dividing the actual uptake rate of a cell by the uptake rate within pure growth medium, provides a good measure if this condition is met. The relative uptake rate for the Monod kinetic is calculated as follows:

(9) $$\text{Relative uptake rate}=\frac{\text{Upt}}{{\text{Upt}}^{\max}\cdot \frac{c_{0}}{c_{0}+K}},$$

where *Upt* is the uptake of a cell, Upt^max^ the maximal uptake of the cell and *c*_0_ the concentration of limiting nutrient within growth medium.

### Colony representations

Experimental data from a colony of *C. glutamicum* growing within the modelled device were used to derive the investigated model geometries (Fig. S2). The process of generating the model geometries is largely automated using matlab® (The MathWorks, 2016) and comsol® via MATLAB link, which connects both programs (Fig. 2A). This is a major improvement over the manual geometry creation used before (Westerwalbesloh *et al*., 2015) as it allows fast and accurate generation of very complex geometries adhering closely to experimental data.

Microscopic pictures of the example colony at five different time points, representing colony sizes of approximately 2, 20, 50, 100 and 200 cells, are analysed using a custom FIJI plugin (Helfrich *et al*., 2015a). The corresponding image for 200 cells is shown in Fig. 1D. For each cell, the position within the chamber, the area and the angle towards the *x*‐axis are determined semi‐automatically. These data are then transferred into matlab®, where the area is used to estimate the biovolume of each individual cell (Section Cell metabolism). Depending on the chosen geometric representation, the cell coordinates and angles are used to place point sources or three‐dimensional objects (Fig. 2B). The centre of the colony, calculated as barycentre weighting each cell equally, is moved to the centre of the chamber to ensure all different colonies are positioned at the same point. The models are described in the following paragraphs; images of cell colonies and models is shown in Fig. S2. The estimated biovolume is used to calculate the metabolic uptake parameters for the mass transfer simulation (Section Mass transfer) and connect metabolism with mass transfer implementing an additional reaction term per volume, a boundary condition on the cell surfaces or point sources, depending on the dimensionality of the cell representation.

#### Colonypoint model (Model 1a)

In this model, a single point source represents the whole colony. The metabolic rate of this point is the sum of all single‐cell uptake rates. In the CFD simulation, a point source is implemented as very small volume of finite dimensions, whose exact size depends on the mesh density.

#### Cellpoint model (Model 1b)

Here, each cell is modelled as individual point source. The position of the points are the positions at the centre of each cell.

#### Colonyvolume model (Model 2a)

The colony is represented as homogeneous volume occupied by a cylinder with a radius equal to the largest distance of a single cell from the chamber centre. The cellular metabolism is implemented by distributing the sum of single‐cell uptake rates over the volume as additional reaction term in the mass transfer equations (Eq. (6)).

#### Colonyvolume model with adjusted diffusion (Model 2b)

This model is similar to Model 2a, but the diffusion coefficient within the volume of the colony is adjusted. This implies that the cells in the chambers reduce the liquid volume available for mass transfer and block the direct diffusion paths, especially for molecules like sugars and amino acids (Villadsen *et al*., 2011). The effective diffusion coefficient *D*′ within the colony volume is calculated using Maxwell's solution for a regular array of cylinders, as the geometry is flat and thereby similar to a two‐dimensional problem (Ochoa‐Tapia *et al*., 1994; Eq. (10)). The three‐dimensional equivalent has already been used to facilitate mass transfer calculations in macroscopic biofilms (Wood *et al*., 2002).

(10) $$\frac{D^{\prime }}{D}=\frac{1-\varepsilon_{\sigma }}{1+\varepsilon_{\sigma }}$$

Here, we assume a biovolume fraction of cylinders with *ε*_*σ*_ = 0.4, which is the volume fraction of cells in a square colony part of 225 μm^2^ in the centre of the colony with 200 cells for Model 3a. *D* denotes the diffusion coefficient of a substance in water.

#### Cellbody model (Model 3a and Model 3b)

The most complex model incorporates the cells as three‐dimensional objects, cylinders with spherical domes at both ends. The diameter of the cylinders and the domes is chosen to be 0.84 μm while the cultivation chamber has a height of 0.8 μm. That is equivalent to cells with flexible walls which touch the floor and the ceiling of the chamber, where the upper and lower parts of the cylinder are cut off horizontally (magnification in Fig. 2B). Lengths of each cell are estimated according to Eq. (1). The model was created for the cell areas as they were recognized by the software (Model 3a) and once for cells which are 25% longer, to show how the cell shape potentially influences mass transfer (Model 3b). The metabolism is evaluated locally on the cell surface; therefore, the uptake rate on a point of the surface depends on the concentration *c* at the point where the equation is evaluated. The calculated uptake rate for the estimated cellular biovolume is divided by the cell surface area within the chamber, so that the parts which are cut off, as they touch the upper or lower surface, are not considered.

## Conflict of interest

The authors have declared no conflict of interest.

## Supporting information


**Table S1.** Number of mesh elements.
**Table S2.** Mesh independence for geometry with 100 cells.
**Fig. S1.** Measurements of the simulated chamber geometry with supply channels.
**Fig. S2.** Different model geometries in comparison to the respective microscope images.
**Fig. S3.** Nutrient distribution within microcolonies based on simulations with different coarse graining concepts.
**Fig. S4.** Nutrient distribution within microcolonies based on simulations with different coarse graining concepts.
**Fig. S5.** The concentrations *B* and *A*–*B* normalized to *C*, the Gini coefficient and the average relative uptake rate for each model and colony size and the nutrient concentration of 0.5 mmol l^−1^.
**Fig. S6.** The concentrations *B* and *A*–*B* normalized to *C*, the Gini coefficient and the average relative uptake rate for each model and colony size and the nutrient concentration of 5 mmol l^−1^.
**Fig. S7.** The concentrations *B* and *A*–*B* normalized to *C*, the Gini coefficient and the average relative uptake rate for each model and colony size and the nutrient concentration of 50 mmol l^−1^.
