# Supplementary material for: Coarse‐graining bacteria colonies for modelling critical solute distributions in picolitre bioreactors for bacterial studies on single‐cell level
Source: Microb Biotechnol. 2017 Apr 3;10(4):845–57. doi: 10.1111/1751-7915.12708 (PMC5481542; doi:10.1111/1751-7915.12708)
Supplement: Supplementary file 1 — Table S1. Number of mesh elements. Table S2. Mesh independence for geometry with 100 cells. Fig. S1. Measurements of the simulated chamber geometry with supply channels. Fig. S2. Different model geometries in comparison to the respective microscope images. Fig. S3. Nutrient distribution within microcolonies based on simulations with different coarse graining concepts. Fig. S4. Nutrient distribution within microcolonies based on simulations with different coarse graining concepts. Fig. S5. The concentrations B and A–B normalized to C, the Gini coefficient and the average relative uptake rate for each model and colony size and the nutrient concentration of 0.5 mmol l−1. Fig. S6. The concentrations B and A–B normalized to C, the Gini coefficient and the average relative uptake rate for each model and colony size and the nutrient concentration of 5 mmol l−1. Fig. S7. The concentrations B and A–B normalized to C, the Gini coefficient and the average relative uptake rate for each model and colony size and the nutrient concentration of 50 mmol l−1. [file MBT2-10-845-s001.pdf]

Supplementary Information

Coarse graining bacteria colonies for modeling critical solute distributions in picoliter bioreactors for bacterial studies on single-cell level

Christoph Westerwalbesloh, Alexander Grünberger,
Wolfgang Wiechert, Dietrich Kohlheyer and Eric von Lieres*

January 23, 2017

Institute of Bio- and Geosciences, IBG-1: Biotechnology,
Forschungszentrum Jülich, Jülich 52425 Germany;
telephone: +49-2461-61-2168; fax: +49-2461-61-3870; e-mail:
e.von.lieres@fz-juelich.de

Table 1: Number of mesh elements

Model	2 Cells	20 Cells	50 Cells	100 Cells	200 Cells
Model 1a	1.3619×10^6	1.3619×10^6	1.3619×10^6	1.3619×10^6	1.3619×10^6
Model 1b	1.3619×10^6	1.3620×10^6	1.3612×10^6	1.3615×10^6	1.3616×10^6
Model 2a	1.3633×10^6	1.3635×10^6	1.3630×10^6	1.3643×10^6	1.3633×10^6
Model 2b	1.3635×10^6	1.3636×10^6	1.3635×10^6	1.3650×10^6	1.3642×10^6
Model 3a	1.5721×10^6	2.9147×10^6	4.3734×10^6	6.8687×10^6	4.7444×10^6
Model 3b	1.5817×10^6	1.7546×10^6	4.4118×10^6	3.1349×10^6	6.4294×10^6

Table 2: Mesh independence for geometry with 100 cells

Model	Elements Normal mesh	Elements Refined mesh	Maximum concentration difference in xy-plane at half-chamber height
Model 1a	1.3619×10^6	6.050076×10^6	4.9×10^{-2} mmol/L
Model 1b	1.3615×10^6	6.052157×10^6	2.1×10^{-3} mmol/L
Model 2a	1.3643×10^6	6.079938×10^6	3.5×10^{-3} mmol/L
Model 2b	1.3650×10^6	6.079938×10^6	3.6×10^{-3} mmol/L
Model 3a	6.8687×10^6	26.281688×10^6	2.7×10^{-3} mmol/L
Model 3b	3.1349×10^6	13.547256×10^6	5.5×10^{-3} mmol/L

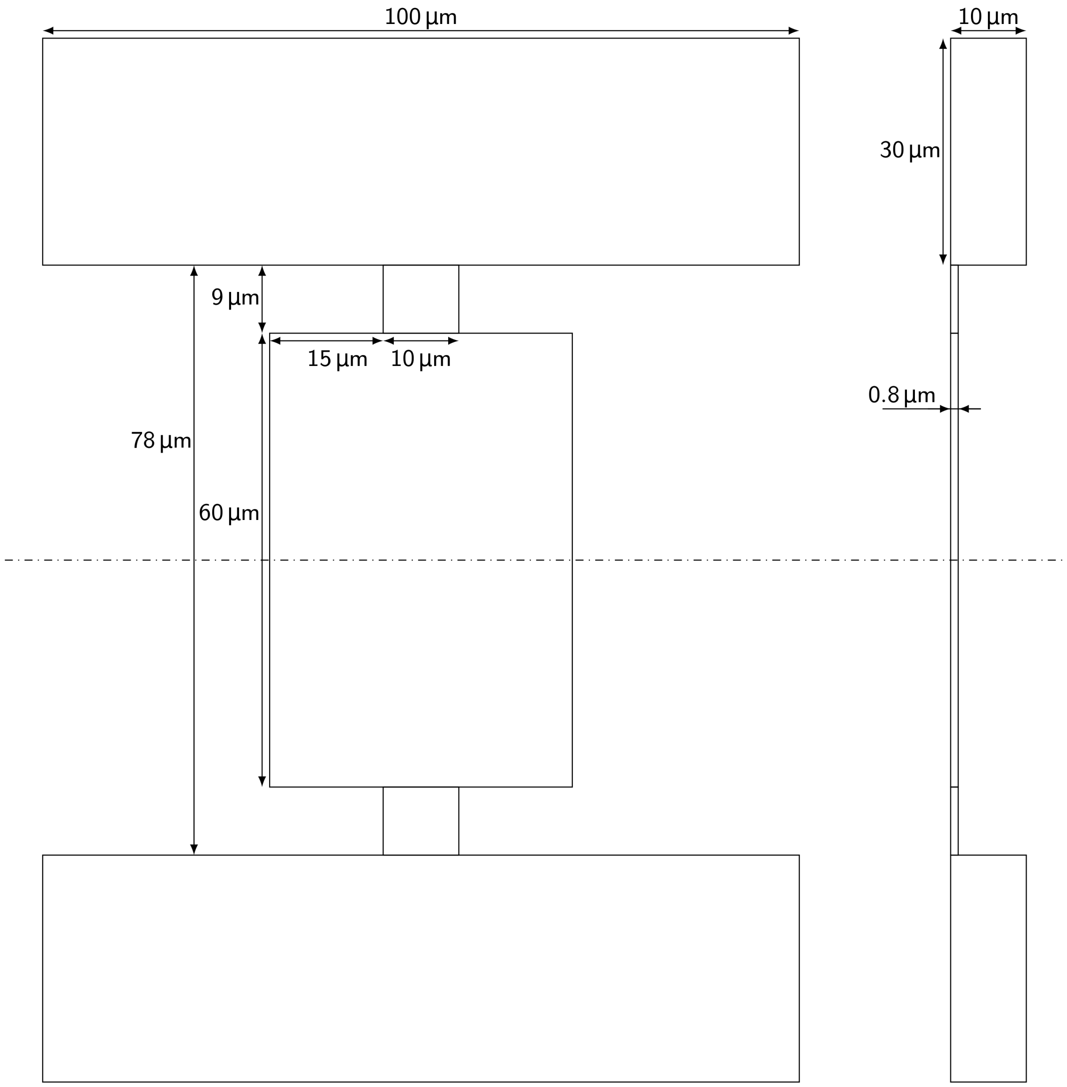

Figure 1: Measurements of the simulated chamber geometry with supply channels.

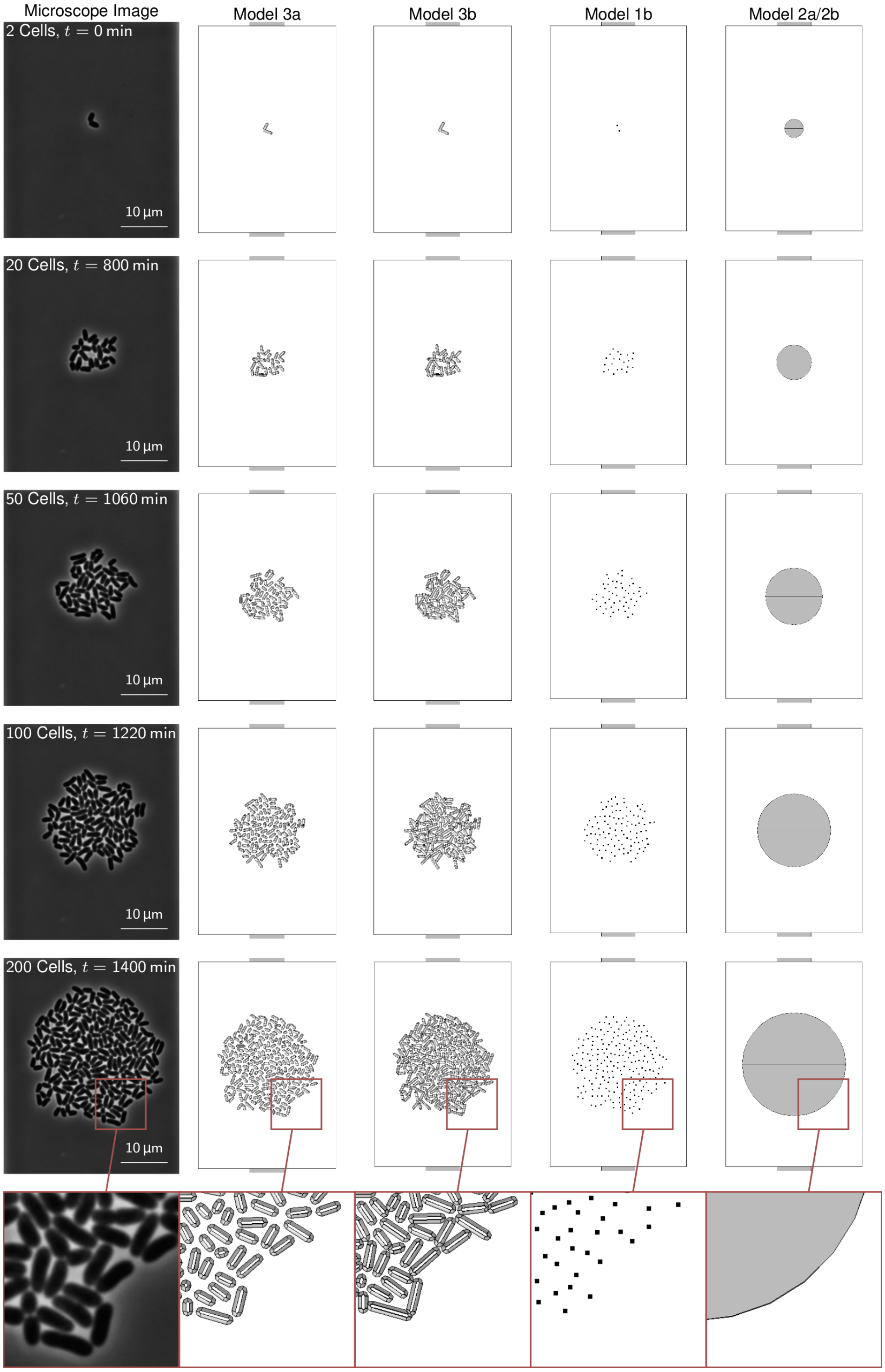

Figure 2: Different model geometries in comparison to the respective microscope images. Model 1a is not shown.

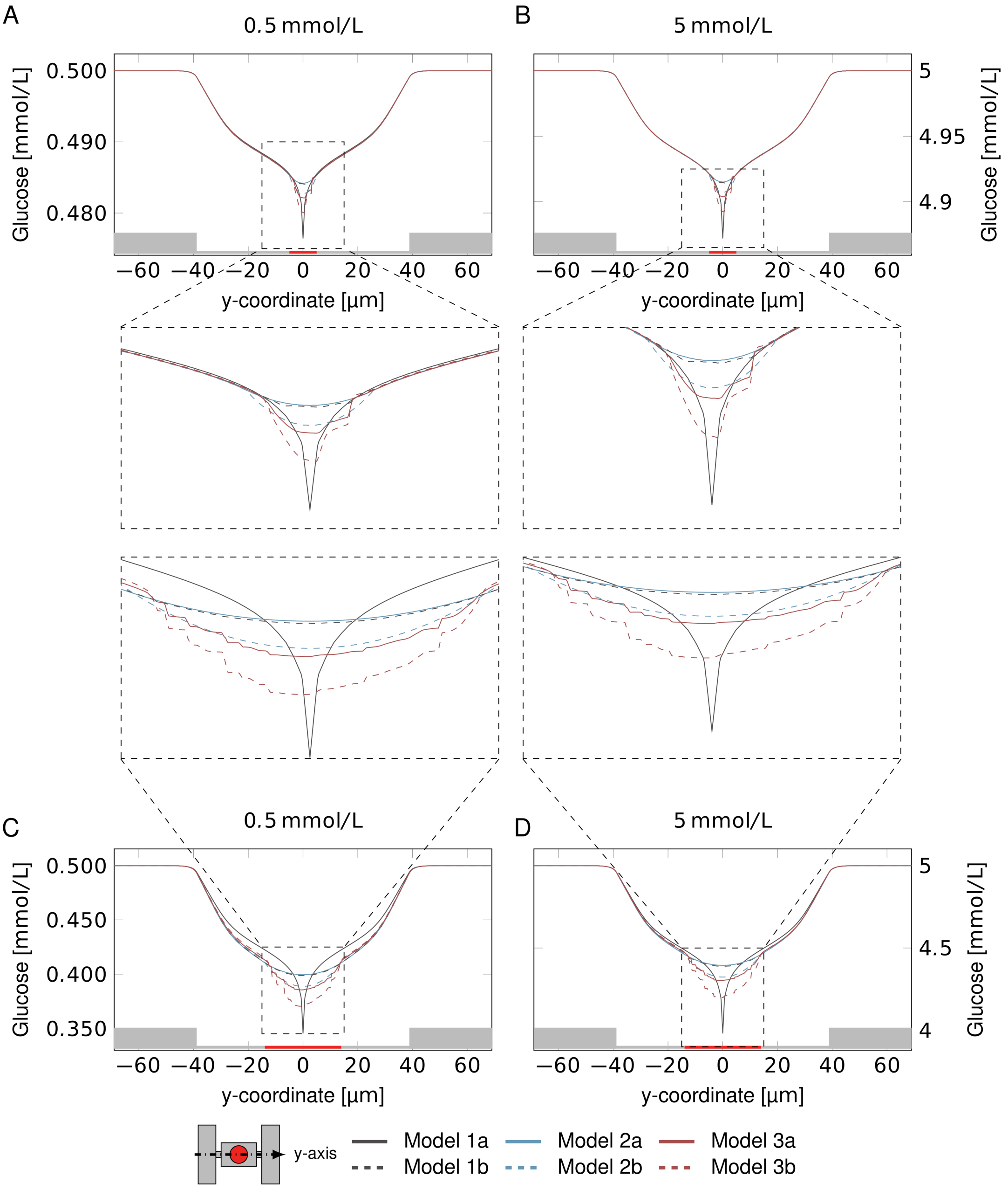

Figure 3: Nutrient distribution within microcolonies based on simulations with different coarse graining concepts. The graphs and respective zoom-outs show the glucose concentration along the y-axis through the center of the chamber/colony in mmol/L for the colony with 20 cells (A, B) and 200 cells (C, D) and two medium glucose concentrations of 0.5 mmol/L (A,C) and 5 mmol/L (B,D).

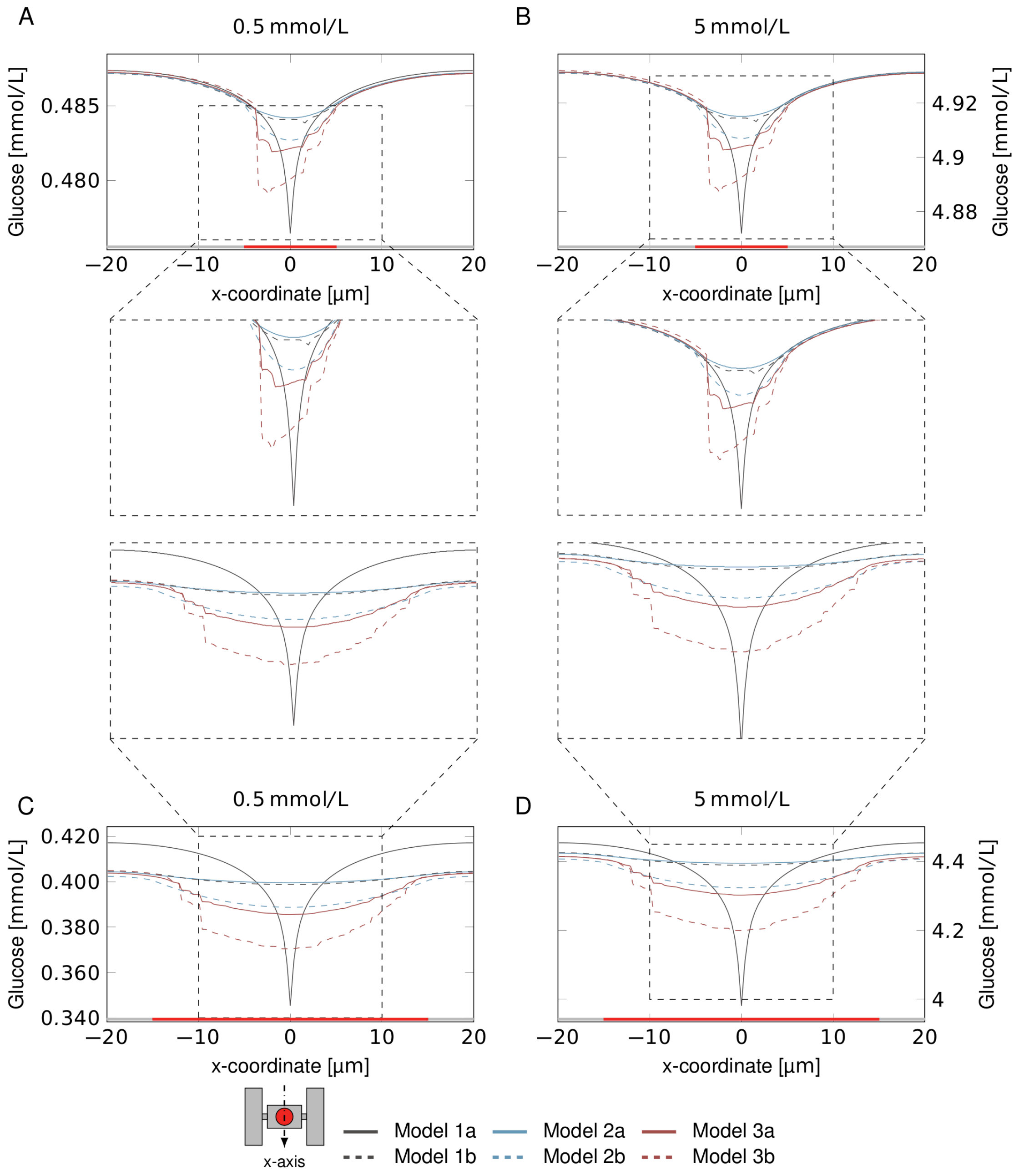

Figure 4: Nutrient distribution within microcolonies based on simulations with different coarse graining concepts. The graphs and respective zoom-outs show the glucose concentration along the x-axis through the center of the chamber/colony in mmol/L for the colony with 20 cells (A, B) and 200 cells (C, D) and two medium glucose concentrations of 0.5 mmol/L (A,C) and 5 mmol/L (B,D).

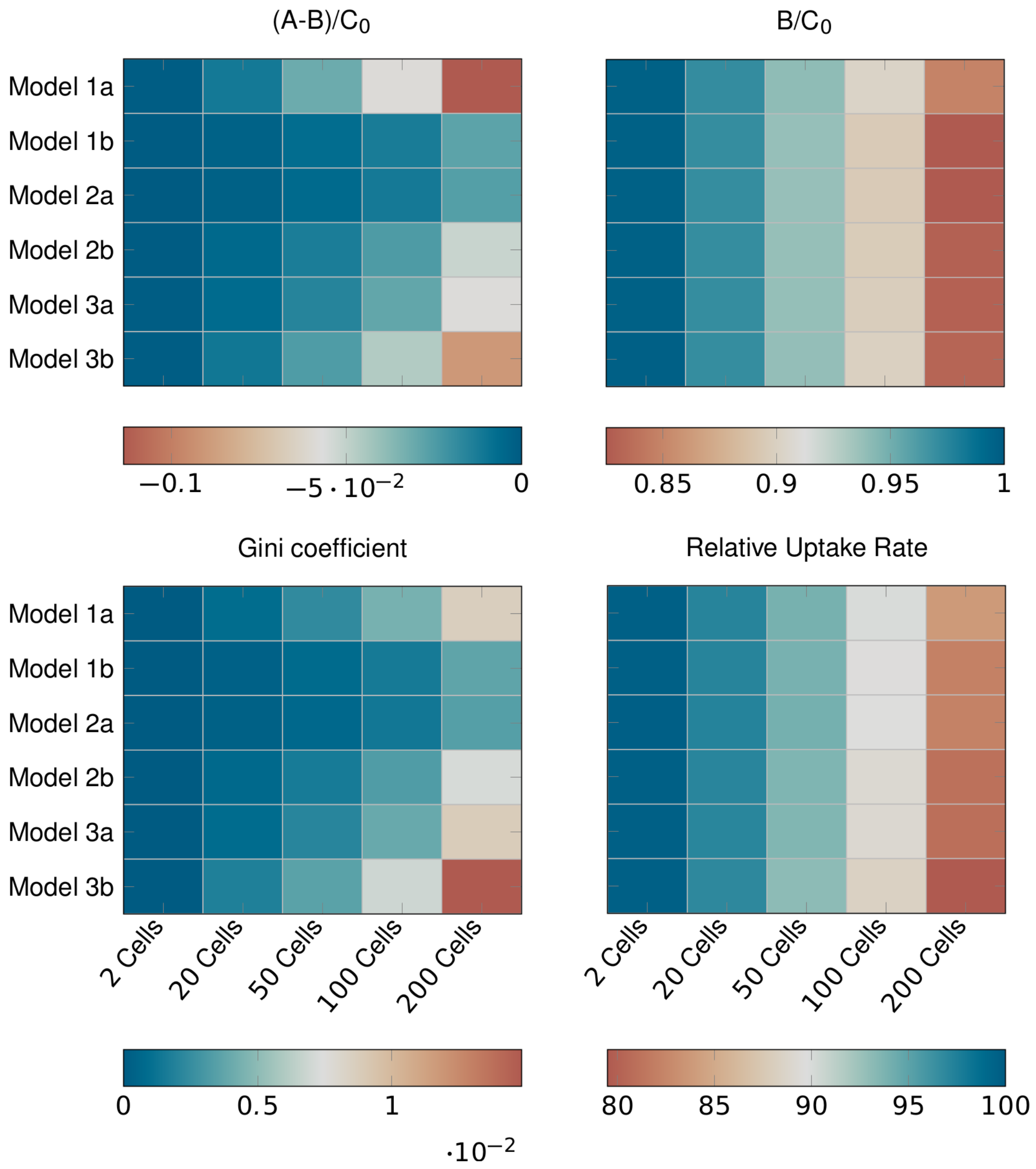

Figure 5: The concentrations B and A − B normalized to C, the Gini coefficient and the average relative uptake rate for each model and colony size and the nutrient concentration of 0.5 mmol/L.

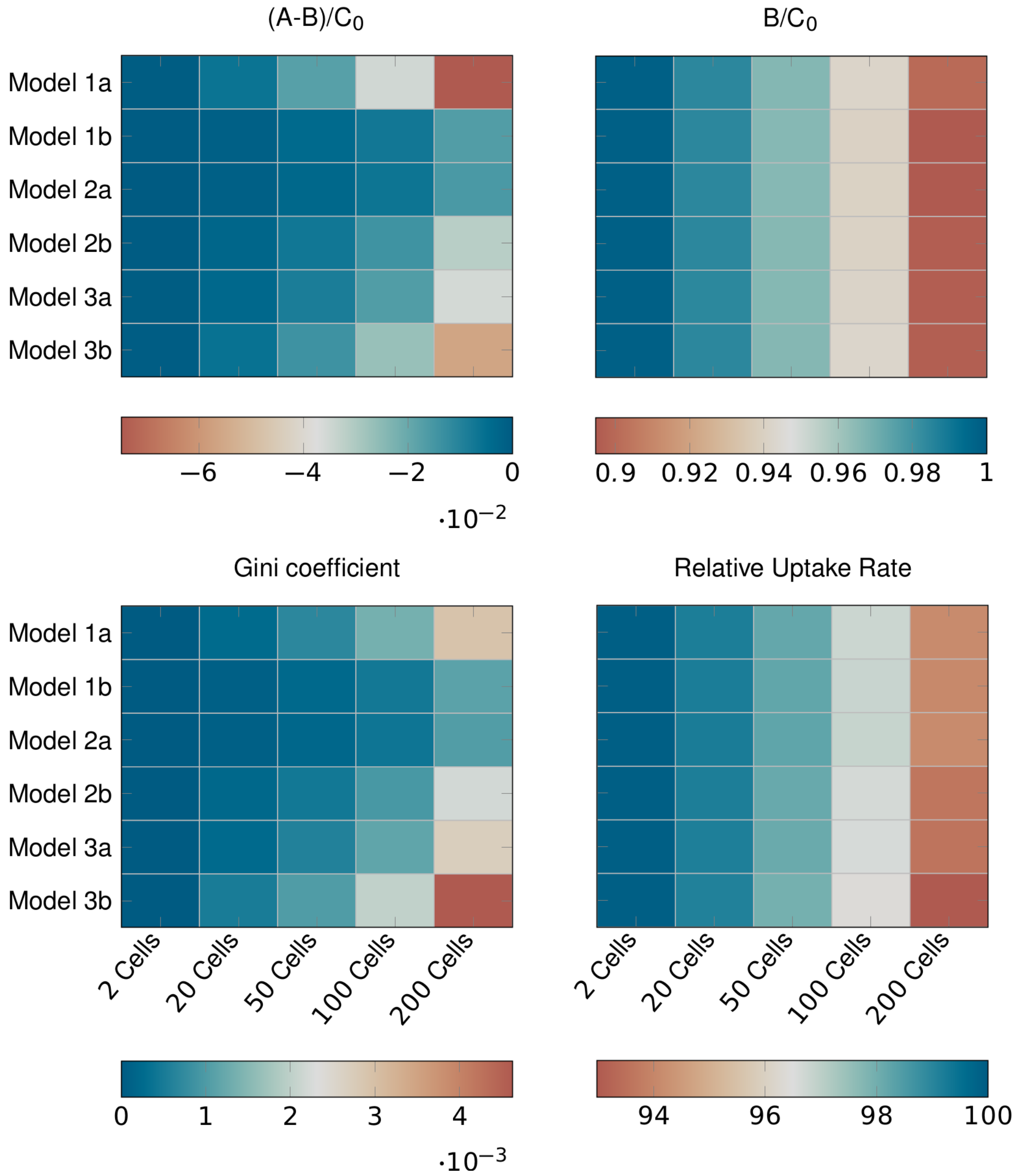

Figure 6: The concentrations B and A − B normalized to C, the Gini coefficient and the average relative uptake rate for each model and colony size and the nutrient concentration of 5 mmol/L.

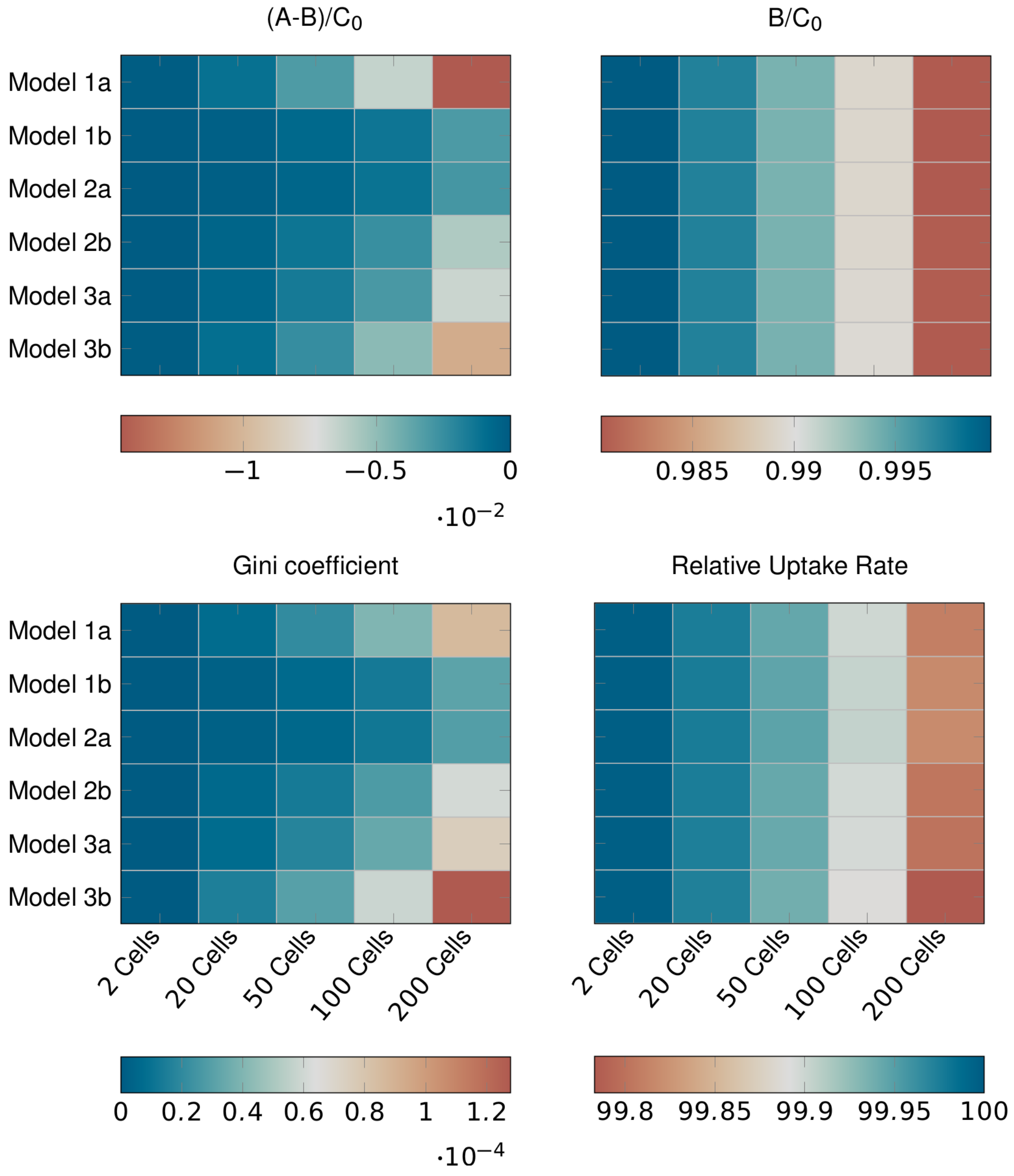

Figure 7: The concentrations B and A − B normalized to C, the Gini coefficient and the average relative uptake rate for each model and colony size and the nutrient concentration of 50 mmol/L.
